# Neuroprotective effects of G9a inhibition and cannabinoid receptor activation in Alzheimer's disease through a pharmacological approach

**DOI:** 10.1016/j.neurot.2025.e00616

**Published:** 2025-05-31

**Authors:** Aina Bellver-Sanchis, Marta Ribalta-Vilella, Jaume Lillo, Daniel Ortuño-Sahagún, Rafael Franco, Mercè Pallàs, Gemma Navarro, Christian Griñán-Ferré

**Affiliations:** a Department of Pharmacology and Therapeutic Chemistry, Institut de Neurociències-Universitat de Barcelona, Avda. Joan XXIII, 27, 08028 Barcelona, Spain; b Facultat de Farmàcia i Ciències de l'Alimentació or Facultat de Biologia (Depending on the Faculty You Are Registered with), Universitat de Barcelona, Avda. Diagonal 643, 08028 Barcelona, Spain; c Network Center for Biomedical Research in Neurodegenerative Diseases, CiberNed, Spanish National Health Institute Carlos III, Av. Monforte de Lemos, 3-5, 28029 Madrid, Spain; d Institut de Neurociències UB, Campus Mundet, Passeig de la Vall d’Hebron 171, 08035 Barcelona, Spain; e Molecular Neurobiology Laboratory, Department Biochemistry and Molecular Biomedicine, Facultat de Biologia, Universitat de Barcelona, 08028 Barcelona, Spain; f Laboratorio de Neuroinmunobiología Molecular, Instituto de Investigación en Ciencias Biomédicas, Departamento de Biología Molecular y Genómica. Universidad de Guadalajara, Guadalajara, Mexico; g Department of Biochemistry and Physiology, School of Pharmacy and Food Sciences, Universitat de Barcelona, 08028 Barcelona, Spain; h School of Chemistry, University of Barcelona, 08028 Barcelona, Spain; i Spanish Biomedical Research Center in Neurodegenerative Diseases (CIBERNED)-Instituto de Salud Carlos III, Madrid, Spain

**Keywords:** Neurodegenerative diseases, Cannabinoids, Histone methyltransferase, Behavior, Neuroinflammation, Synaptic plasticity

## Abstract

Epigenetic alterations are key contributors to Alzheimer's disease (AD), driving age-related cognitive decline. This study explores the combined neuroprotective effects of G9a histone methyltransferase inhibition (via UNC0642) and cannabinoid receptor activation (CB1R: ACEA; CB2R: JWH133) in AD models. We used HEK-293T cells and hippocampal neurons to demonstrate that G9a inhibition selectively enhances CB1R-mediated ERK/cAMP signaling. In SAMP8 mice (sporadic AD model), we evaluated the effects of pharmacological inhibition of G9a (UNC0642), combined with CB_1_R agonism (ACEA) and/or CB_2_R agonism (JWH133), on cognitive recovery, neuronal morphology, and neuroinflammation. Our results demonstrated that SAMP8 mice treated with UNC0642 and ACEA exhibited significant recovery in short-term memory, as assessed by the Novel Object Recognition Test (NORT), and complete recovery of spatial memory in the Object Location Test (OLT). These improvements were accompanied by enhanced neuronal morphology (increased dendritic length and density) and reduced neuroinflammation markers, suggesting a synergistic effect of G9a inhibition and CB_1_R activation. Importantly, JWH133 treatment, both alone and in combination with UNC0642, resulted in a pronounced reduction of neuroinflammatory markers (*Trem2, Cd33, iNOS*) and a significant restoration of dendritic spine density and branching length, with the dual treatment showing the most robust effects. JWH133 alone produced moderate cognitive improvement, but its combination with G9a inhibition led to outcomes comparable to those of control animals. Thus, the results underscore G9a inhibition's potential to amplify cannabinoid receptor-mediated neuroprotection while mitigating psychoactive risks, offering a promising multi-target approach for neurodegenerative diseases.

## Graphical abstract

**Figure:**
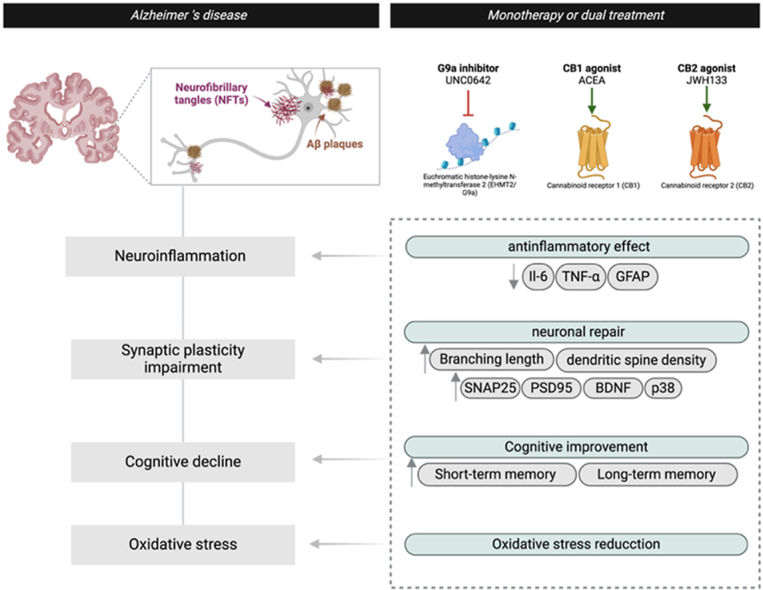

## Introduction

Alzheimer's disease (AD) is a debilitating neurodegenerative disorder that poses one of the most significant challenges to modern medicine [1]. AD is characterized by progressive cognitive decline and is associated with the accumulation of amyloid-beta (Aβ) plaques and neurofibrillary tangles (NFTs), synaptic dysfunction and neuronal loss [2]. The complexity of AD pathology has led researchers to explore a variety of therapeutic targets, of which the endocannabinoid system (ECS) and epigenetic mechanisms have received considerable attention [[3], [4], [5]].

The ECS, consisting of endogenous cannabinoids, such as anandamide (AEA) and 2-arachidonoylglycerol (2-AG), their receptors (CB_1_R and CB_2_R), and metabolic enzymes, plays a pivotal role in the maintenance of neuronal homeostasis [6]. CB_1_Rs are mainly expressed in the central nervous system (CNS) and are involved in modulating the release of neurotransmitters. CB_2_Rs, which are found primarily in peripheral tissues, are expressed in glial cells, especially following activation due to neuroinflammation [7]. The ECS is disrupted in AD, and modulation of cannabinoid receptors has emerged as a potential therapeutic strategy to mitigate the progression of pathology and symptoms associated with AD. Several synthetic cannabinoids, such as JWH133, a selective agonist CB_2_R, and ACEA, a selective agonist CB_1_R, have been investigated for their potential therapeutic effect in AD [8,9]. Despite these promising results, the use of cannabinoids in AD therapy is not without limitations. The psychoactive effects of THC and the potential impairment of memory and learning ability associated with the use/abuse of cannabis in vulnerable individuals are concerns that need to be addressed. Additionally, the optimal therapeutic dosage and timing of treatment have yet to be determined.

In parallel with research into the ECS, epigenetic changes have been recognized as key factors in the pathogenesis of AD [10,11]. Epigenetic mechanisms, such as DNA methylation and histone modifications, regulate gene expression without altering the underlying DNA sequence. These reversible modifications respond to environmental stimuli and can influence the course of neurodegenerative diseases [10,12]. G9a, also known as EHMT2, is a histone methyltransferase that primarily catalyzes the di-methylation of histone H3 at lysine 9 (H3K9me2), a marker generally associated with gene silencing [13]. G9a is related to various biological processes, including development, differentiation, and the response to neuronal injury [14]. In the context of AD, the role of G9a in gene regulation is of particular interest as it may affect genes involved in neurodegeneration and memory formation [15]. UNC0642 is a potent and selective G9a inhibitor developed as a tool to study biological function and is a potential therapeutic target for AD [5,[16], [17], [18], [19], [20], [21], [22], [23]].

The interplay between G9a, histone modifications, and cannabinoid receptor expression reveals a compelling epigenetic dimension to the dysregulation of the endocannabinoid system (ECS) observed in AD. Rather than resulting from genetic mutations, these changes arise from alterations in gene expression-specifically, G9a-mediated histone methylation that represses CB1 receptor (CB1R) transcription. G9a catalyzes the dimethylation of histone H3 at lysine 9 (H3K9me2), a repressive mark that silences the *CNR1* gene, leading to reduced CB1R expression in affected brain regions and dorsal root ganglia (DRG) [[24], [25], [26]]. Since CB1R is crucial for learning and memory, its suppression by G9a may underlie some of the cognitive deficits characteristic of AD [25,27]. In contrast, studies have shown that after nerve injury, CB2 receptor (CB2R) mRNA levels are elevated in the DRG, a response associated with increased activating histone marks (H3K4me3, H3K9ac) and decreased repressive marks at the *CNR2* promoter [24,28]. Given that G9a is generally linked to gene silencing, it is unlikely to be directly responsible for CB2R upregulation. This differential regulation of cannabinoid receptors highlights a nuanced and dynamic relationship between epigenetic mechanisms and receptor expression in the nervous system's response to injury and disease, underscoring the importance of epigenetic factors in ECS dysfunction and pointing to new avenues for therapeutic intervention in AD [24,25,29].

Moreover, the potential role of G9a in modulating neuroinflammatory responses by regulating CB_2_R expression is another area of interest. Since neuroinflammation is a hallmark of AD and CB_2_Rs are involved in the modulation of immune responses, epigenetic regulation of CB_2_Rs by G9a could influence the progression of AD [30]. Therefore, targeting G9a in AD holds considerable therapeutic potential. Modulation of G9a could restore the expression of cannabinoid receptors and other dysregulated genes, potentially reversing or slowing AD progression. This could improve the efficacy of cannabinoid-based therapies, which show promise but are limited by the complexity of the ECS. In addition, studying G9a in AD could provide insights into the broader epigenetic landscape and support the identification of new biomarkers and therapeutic targets. Epigenetic therapies could provide personalized treatment options, underscoring the importance of understanding the interactions of G9a with other epigenetic modifiers.

In this study, we investigated the neuroprotective potential of the G9a inhibitor UNC0642 through in vitro and in vivo experiments, assessing its effects on molecular and pathological markers of AD. We also evaluated the therapeutic potential of synthetic agonists of cannabinoid receptors, JWH133 and ACEA, individually and in combination with UNC0642, in the SAMP8 mouse model of accelerated senescence. By integrating G9a inhibition with cannabinoid receptor activation, this work aims to elucidate the interplay between epigenetic regulation and endocannabinoid signaling, providing insights into novel therapeutic strategies for AD.

## Materials and methods

### Cell culture and transient transfection

HEK-293T cells were grown in Dulbecco's modified Eagle's medium (DMEM) (15-013-CV, Corning, NY, USA) supplemented with 2 ​mM L-glutamine, 100 U/ml penicillin/streptomycin, MEM Non-Essential Amino Acids Solution (1/100) and 5 ​% (v/v) heat-inactivated Fetal Bovine Serum (FBS) (Invitrogen, Paisley, Scotland, UK). Cells were maintained in a humidified atmosphere with 5 ​% CO_2_ at 37 ​°C.

HEK-293T cells grown in 6-well dishes were transiently transfected using the PEI method (PolyEthylenImine, Sigma-Aldrich, St. Louis, MO, USA). The cells were incubated for 4 ​h with the corresponding cDNAs, PEI (5.47 ​mM in residual nitrogen), and 150 ​mM NaCl in the serum-deprived medium. The medium was then replaced with a fresh, complete culture medium, and the cells were incubated for 48 ​h before the experimental procedures.

To prepare primary neuronal cultures, the brain was removed from C57/BL6 mice embryos (E19). The hippocampal was dissected, and the meninges were carefully removed and digested with 0.25 ​% trypsin at 37 ​°C for 20 ​min. Trypsinization was stopped by washing the tissue. Cells were transferred to a cell suspension by passage through 0.8 ​mm and 0.5 ​mm needles, followed by passage through mesh with 100 ​μm pores. Neurons were resuspended in medium and plated at a density of 1 ​× ​106 ​cells/ml in 6-well dishes in neurobasal medium (21103-049, Gibco, Paisley, Scotland, UK), supplemented with 2 ​mM L-glutamine, 100 U/mL penicillin/streptomycin, a MEM preparation of non-essential amino acids (1/100), and 2 ​% (v/v) B27 supplement (17504-044, Gibco, Paisley, Scotland, UK). Cultures were maintained for 12 days at 37 ​°C in a humidified atmosphere with 5 ​% CO_2_.

### cAMP levels determination

Two hours before initiating the experiment, transfected HEK-293T cells or neuronal primary cultures medium was replaced with, respectively, serum-free DMEM or neurobasal mediums. Cells were then detached, resuspended in DMEM serum-free medium containing 50 ​μM zardaverine, and plated in 384-well microplates (2500 ​cells/well). Cells were pretreated with G9a inhibitor or vehicle (15 ​min) and stimulated with selective agonists (15 ​min) before adding 0.5 ​μM Forskolin to increase cAMP levels. 15 min later, 500 ​nM fluorophore-containing ULight™ antibody (5 ​μl) and cAMP-Europium (cAMP-Eu) (5 ​μl) were added. Incubation was prolonged for 1 ​h at 25 ​°C, and the PHERAstar Flagship reader equipped with an HTRF optical module (BMG Lab Technologies, Offenburg, Germany) was used for measuring the 665/620 ​nm ratio.

### Extracellular signal-regulated kinase phosphorylation assays

To determine the phosphorylation of extracellular signal-regulated kinase 1/2 (ERK1/2), transfected HEK-293T cells were grown in 96-well plates. On the day of the experiment, the medium was replaced with serum-free medium 2 ​h before initiating the experiment. Cells were pretreated at 25 ​°C for 10 ​min with the G9a inhibitor or vehicle and stimulated for 7 ​min with selective agonists. Then, cells were washed twice with cold PBS before adding the lysis buffer (15 ​min treatment in agitation). Thereafter, 10 ​μL of the supernatant was added to each well in a white ProxiPlate 384-well plate, and ERK 1/2 phosphorylation was determined using an AlphaScreen®SureFire® kit (PerkinElmer) according to the manufacturer's instructions. Readings were recorded using an EnSpire® Multimode Plate Reader (PerkinElmer, Waltham, MA, USA). The reference value (100 ​%) was determined in the absence of any treatment (basal). The effect of the ligands was expressed as a percentage of the baseline value.

### Animals

**Fig. 1:**
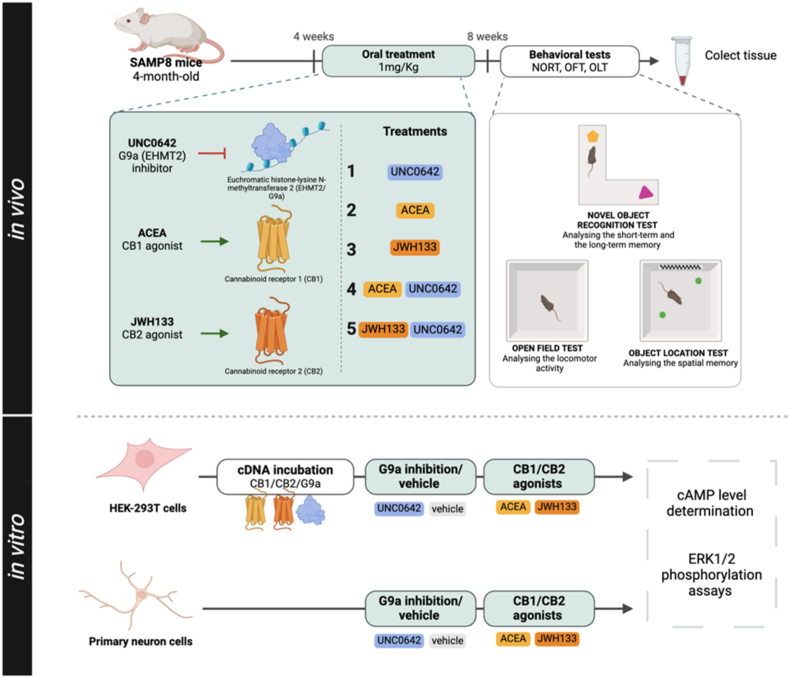
Scheme of experimental design.

6-month-old SAMR1 and SAMP8 male mice (n ​= ​62) were used for behavioral and molecular studies. Animals had free access to food and water and were maintained under standard temperature conditions (22 ​± ​2 ​°C), controlled humidity, and a 12-h light/dark cycle (300 lx/0 lx). SAMP8 animals were randomly assigned to the following groups: SP8 Ct (treated with vehicle, n ​= ​10), SP8 UNC0642 (treated with 1 ​mg/kg G9a inhibitor, n ​= ​7), SP8 ACEA (treated with 1 ​mg/kg CB_1_R agonist, n ​= ​8), SP8 JWH133 (treated with 1 ​mg/kg CB_2_R agonist, n ​= ​8), SP8 ACEA ​+ ​UNC0642 (treated with 1 ​mg/kg CB_1_R agonist and 1 ​mg/kg G9a inhibitor, n ​= ​9) and SP8 JWH133 ​+ ​UNC0642 (treated with 1 ​mg/kg CB_2_R agonist and 1 ​mg/kg G9a inhibitor, n ​= ​8) (Fig. 1). The animals were weighed weekly. The experimental groups received either a daily dose of vehicle (1 ​% DMSO (Dimethyl sulfoxide) (Sigma-Aldrich, Steinheim, Germany, #D4540), 20 ​% w/v, (2-hydroxypropyl)-β-cyclodextrin) (Atomole Scientific Co. Ltd., Wuhan, Hubei, China, #AT-20762) or a dose of 1 ​mg/kg per day of each drug group dissolved in vehicle, via oral gavage during the treatment period. After 4 weeks of treatment, behavioral and cognitive tests were performed. During this time and until sacrifice, the mice continued to receive the single or combination treatment or vehicle. All procedures involving animals, including behavioral tests and dissection and removal of brains, followed ARRIVE and standard ethical guidelines (Council European Communities Directive 2010/63/EU and Guidelines for the Care and Use of Mammals in Neuroscience and Behavioral Research, National Research Council 2003) and were approved by the Institutional Animal Care and Generalitat de Catalunya (#10291, January 28, 2018). Every effort was made to minimize the number of mice used and their suffering.

### Behavioral and cognitive tests

#### Object location test (OLT)

The OLT was performed to investigate the spatial memory of mice when exposed to a new location of an already-known object. This test is based on the spontaneous tendency of mice to spend more time exploring a new object than exploring a known object and location to recognize when an object has been misplaced. The test was conducted in a wooden box (50 ​× ​50 ​× ​25 ​cm), in which three of the walls were white, and the last one was black. The animals were habituated to the empty open field arena for 10 ​min on the first day. On the second day, two identical objects were placed in front of the black wall, equidistant from each other and the wall. The objects were 10 ​cm high. The animals were brought into the open field arena and allowed to explore the objects and the surroundings for 10 ​min. Afterward, the animals were returned to their home cages, and the OLT device was cleaned with 70 ​% ethanol. On the third day, an object was placed in front of the opposite white wall to assess the spatial performance of the mice. The trials were recorded with a camera mounted above the open field area. The total exploration time was determined by counting the amount of time (in seconds) spent sniffing the object in the new location (TN) and the object in the old location (TO). The discrimination index (DI) defined as (TN ​− ​TO)/(TN ​+ ​TO), was calculated to assess cognitive performance.

#### Novel object recognition test (NORT)

Short- and long-term memory was analyzed with NORT. The test was performed in a 90°, two-armed, 25-cm-long, and 20-cm-high black maze. The light intensity in the center of the field was 30 lux. The objects to be discriminated were plastic figures (object A, 5.25-cm-high; and object B, 4.75-cm-high). The mice were acclimatized to the device for 10 ​min on each of 3 consecutive days. On day 4, they underwent a 10-min acquisition trial (habituation phase), in which they were placed in the maze and allowed to explore two identical novel objects (A ​+ ​A or B ​+ ​B) at the end of each arm. To avoid a bias in object preference, objects A and B were counterbalanced so that half of the animals in each experimental group were initially confronted with object A and the other half with object B. Object C was used as the new object for both groups for the analysis of long-term memory. The 10-min retention trials (test phase) took place 2 ​h (short-term; A ​+ ​B or B ​+ ​A) and 24 ​h (long-term; A ​+ ​C or B ​+ ​C) after the habituation phase. During the test phase, one of the two identical objects was replaced by a new one, and the time spent exploring the new object (TN) and the old object (TO) was measured manually. The discrimination index (DI) was calculated as (TN ​− ​TO/TN ​+ ​TO). Sniffing or touching objects with the nose and forepaws was considered exploration. The maze, surface, and objects were cleaned with 70 ​% ethanol between trials to eliminate olfactory cues.

### Biochemical experiments

#### Protein levels determination by western blotting (WB)

For WB, tissue samples were homogenized in a lysis buffer containing phosphatase and protease inhibitors (Cocktail II, Sigma-Aldrich, St. Louis, MO, USA), and protein concentration was determined by Bradford's method. Aliquots of 15 ​μg of protein samples from different groups were separated by Sodium dodecyl sulfate-polyacrylamide gel electrophoresis (SDS-PAGE) (8–14 ​%) and transferred onto polyvinylidene difluoride membranes (PVDF, Millipore). Afterward, membranes were blocked in 5 ​% Bovine Serum Albumin (BSA) in 0.1 ​% Tris-buffered saline with Tween 20 (TBS-T) for 1 ​h at room temperature, followed by overnight incubation at 4 ​°C with the primary antibodies presented in Supplementary Table 1.

The next day, membranes were washed 3 times for 5 ​min with Tris-buffered saline with Tween® 20 Detergent (TBS-T) and incubated with secondary antibodies (mouse or rabbit) for 1 ​h at room temperature. Chemiluminescence-based detection was used to view the immunoreactive proteins, following the manufacturer's protocol (ECL Kit, Millipore). Then, digital images were acquired using an Amersham Imager 680, and semiquantitative analyses were performed using ImageLab Software (BioRad, Hercules, CA, USA). Finally, results were expressed in Arbitrary Units (AU), considering the WT control mice group as 100 ​%. Protein loading was routinely monitored by immunodetection of GAPDH. In the same way, for the phosphorylated protein ratio, normalization against GADPH was done before the ratio value calculation.

### Quantitative real-time PCR

Total RNA isolation was carried out using TRIsure™ reagent following the manufacturer's instructions (Bioline Reagent, London, UK). RNA content in the samples was measured at 260 ​nm, and the purity of the samples was determined by the A260/280 and A260/230 ratio in a NanoDrop™ ND-1000 (Thermo Scientific). Reverse transcription-Polymerase chain reaction (RT-PCR) was performed as previously described, to quantify the mRNA expression. Real-time PCR (qPCR) was performed with the StepOnePlus™ Real-Time PCR System (Applied Biosystems™, Vilnius, Lithuania, #4376600), using the SYBR Green PCR Master Mix (Applied Biosystems™, Vilnius, Lithuania, #K0253). Briefly, each reaction mixture consisted of 6.75 ​μL of cDNA (at a concentration of 2 ​μg), 0.75 ​μL of each primer (at a concentration of 100 ​nM), and 6.75 ​μL of SYBR Green PCR Master Mix (2×). The comparative cycle threshold (Ct) method (ΔΔCt) was used to analyze the data, using the housekeeping gene (β-actin) level to normalize for differences in sample loading and preparation. The primers are listed in Supplementary Table 2. Each sample (n ​= ​3–4) was analyzed in duplicate, and the results represented the *n*-fold difference in transcript levels between the different groups.

### Golgi staining, dendritic length, and spine density

The SAMP8 mice were euthanized by cervical dislocation, and the whole brain was removed from the skull (n ​= ​3). Then, the Golgi staining protocol was performed using the FD Rapid GolgiStain™ Kit (D Neurotechnologies, Inc., #PK401) according to the manufacturer's instructions. Images of neurons for dendritic branching analysis were captured using a Leica Thunder microscope (Leica Thunder Imager; Leica Microsystems) at a 40× magnification objective. Neurite length and neuron complexity were measured using NeuronJ macros and Advanced Sholl Analysis. The number of intersections (branch points) within concentric circles with a radius of 10 ​μm was calculated and compared between groups. Images of neurons for spine density analysis were taken at 63× magnification of the oil objective. Spine density was expressed as the number of spines per 50 ​μm dendrite at a maximum distance of 150 ​μm from the soma.

### Statistical analysis

Data analysis was performed using GraphPad Prism Version 9 software (GraphPad Software, San Diego, CA, USA). The group size differed varied depending on the power analysis and the expertise of the authors. Statistical analysis was performed for studies where the size of each group was n ​= ​5–6 samples per group for in vivo studies and n ​= ​3–4 replicates for in vitro studies. Results were expressed as the mean ​± ​standard error of the mean (SEM). A normality test was performed to ensure that parametric tests could be used. Means were compared using one-way ANOVA followed by Tukey's *post hoc* analysis. A comparison between groups was also performed using a two-tailed Student's *T*-test for independent samples. Statistical significance was assumed if the p-values were <0.05. Statistical outliers were identified using Grubbs' test and were removed from the analysis. A blinded analysis was performed for behavioral tests.

## Results

### G9a histone methyltransferase selectively modulates CB_1_ receptor signaling while leaving CB_2_ receptor activation unaffected

**Fig. 2:**
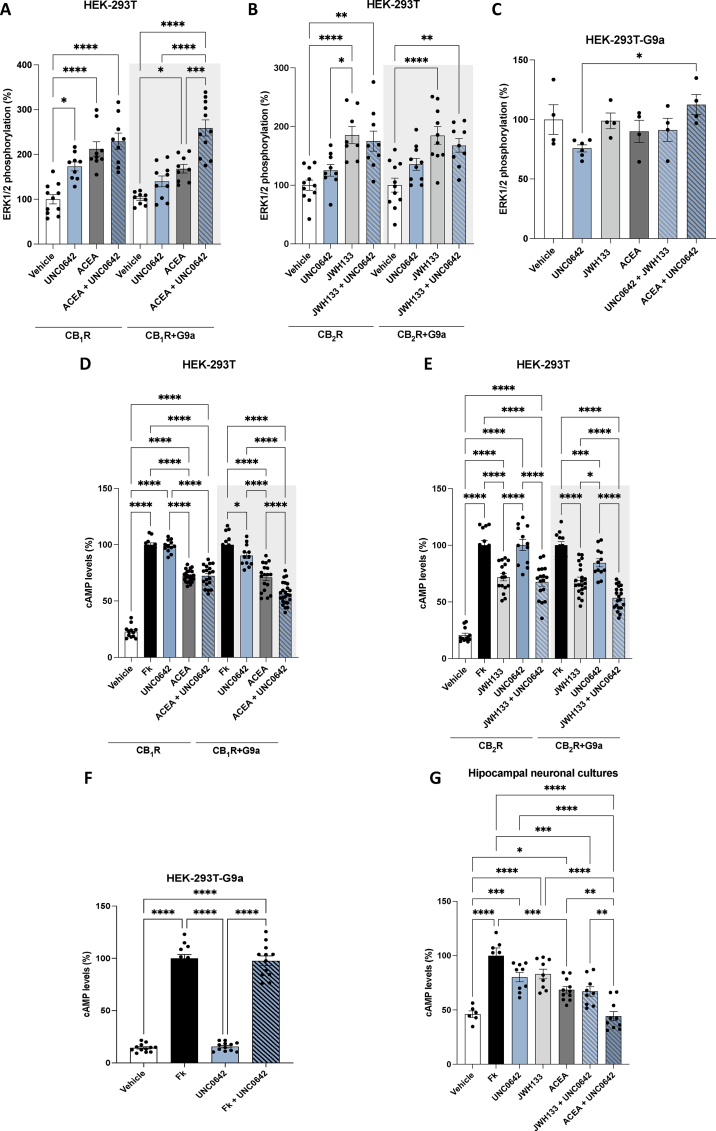
**ERK1/2 phosphorylation and cAMP level determination assays in HEK-293T cells expressing CB_1_R, CB_2_R, in the presence or in the absence of G9a.** HEK-293T cells were transfected with the cDNAs for CB_1_R or CB_2_R in the presence or in the absence of G9a enzyme. Cells were then stimulated with the CB_1_R selective agonist ACEA ot the selecteive CB_2_R agonist JWH-133 in the presence or in the absence of the G9a inhibitor UC0642. Then, MAPK phosphorylation (**A-C**) or cAMP levels (**D-G**) were analyzed. In **D-G** cells were treated for 15 ​min with 500 ​nM forskolin (FK, see Methods) after addition of agonists or UC0642. Values are the mean ​± ​S.E.M. of 6 independent experiments performed in triplicates. One-way ANOVA followed by Bonferroni's multiple comparison post hoc test was used for statistical analysis (∗p ​< ​0.05, ∗∗p ​< ​0.01, ∗∗∗p ​< ​0.001, ∗∗∗∗p ​< ​0.0001).

The HEK-293T-cell-based heterologous system was used to express the human versions of the cannabinoid receptors (CB_1_R or CB_2_R), and the G9a enzyme. On the one hand, the degree of ERK1/2 phosphorylation (*p*-ERK1/2) was measured to assess the activation of the mitogen-activated protein kinase (MAPK) signaling pathway. In cells expressing only CB_1_R, the selective agonist ACEA promoted ERK1/2 phosphorylation, which was further increased in the presence of UNC0642, the G9a inhibitor, thus suggesting that G9a blockade can regulate CB_1_R activation. Moreover, this result is evidence of the endogenous expression of G9a in HEK-293T cells. The modulatory effect of UNC0642 was increased in cells transfected with the plasmids encoding for G9a and CB_1_R (Fig. 2A). In cells only transfected with CB_2_R, the selective agonist, JWH133, increased the levels *p*-ERK1/2 that were not further enhanced by the treatment with the G9a inhibitor. The results were similar in cells transfected with the plasmids encoding for G9a and CB_2_R (Fig. 2B). The effect of UNC0642 on CB_1_R-mediated signaling was specific as it was not found in cells that were only expressing the vector coding for the enzyme (Fig. 2C). Together, these results suggest that G9a could positively regulate CB_1_R activity without affecting the coupling of CB_2_R to the MAPK signaling pathway.

A similar approach was undertaken to check the effect of G9a inhibition on the levels of a second messenger, cAMP, that is regulated by CBR activation. Both cannabinoid receptors are coupled to G_i_ protein, and therefore, their activation reduces the cAMP levels previously increased by 500 ​nM forskolin (FK), an activator of adenylate cyclase. ACEA and JWH133 selective agonists reduced cAMP levels in cells expressing CB_1_ or CB_2_ receptors, respectively, and treated with FK, while the G9a inhibitor had no notable effect (Fig. 2D and E). However, when the enzyme was transfected together with the CB_1_R or CB_2_R plasmids, the G9a inhibitor induced a potentiation of both cannabinoid receptors mediated signalling (Fig. 2D). As expected, in HEK-293T cells transfected only with the plasmid coding for G9a and treated with FK, the cAMP levels were not affected by either the selective cannabinoid agonists or the G9a inhibitor (Fig. 2F). Finally, cAMP levels were evaluated in FK-treated primary hippocampal neurons. Interestingly, the G9a inhibitor enhanced the effect of ACEA, the selective CB_1_R agonist, and also the effect of JWH-133, the selective CB_2_R agonist (Fig. 2G). Therefore, we demonstrate here that a G9a inhibitor can enhance CB_1_R activity in a heterologous system as well as in primary neuronal cultures and does not impair CB_2_R activity in transfected HEK nor in hippocampal neurons.

### Differences in the expression of CB receptor, of microglial activation markers and of proinflammatory cytokines after G9a inhibition and cannabinoid receptor activation in SAMP8 animals

**Fig. 3:**
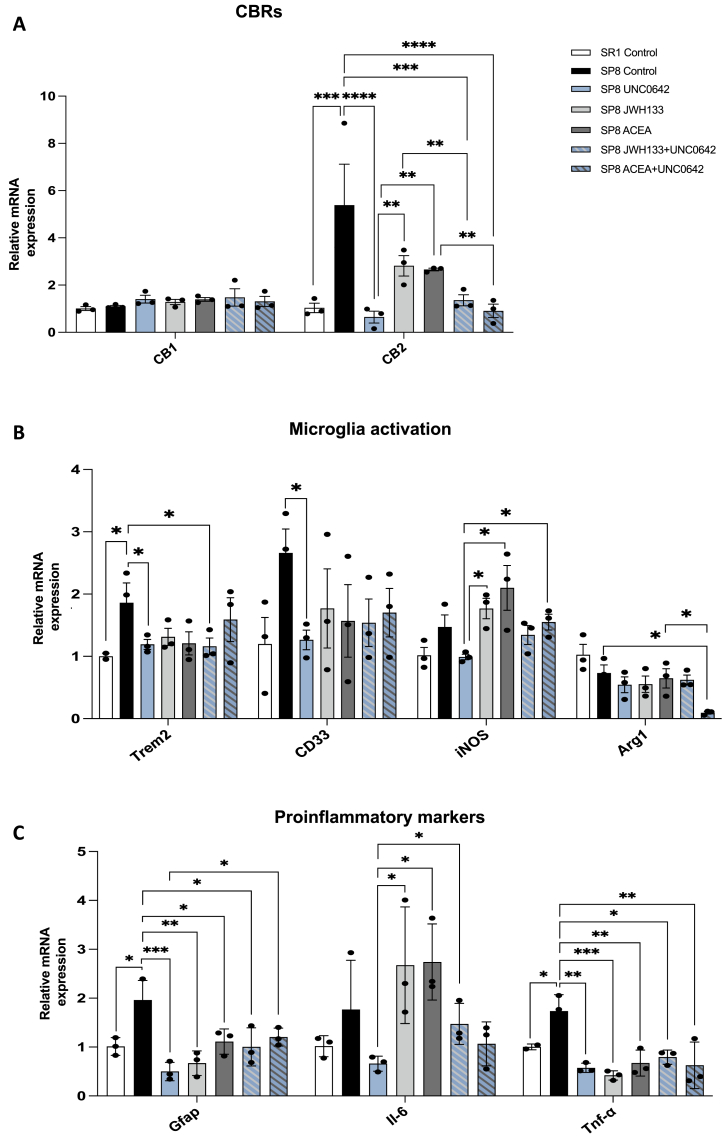
**Relative expression of transcripts coding for CBRs (A), for microglial activation markers (B) and for proinflammatory molecules (C).** mRNa isolation from hippocampi and qPCR assays are detailed in Methods. Samples were from 6-month-old SAMR1 untreated (n ​= ​3), SAMP8 untreated (n ​= ​3) and SAMP8 treated with UNC0642 (n ​= ​3), JWH133 (n ​= ​3), ACEA (n ​= ​3), UNC0642 ​+ ​JWH133 (n ​= ​3) or UNC0642+ACEA (n ​= ​3). Dose was 1 ​mg/kg for the single treatment and 1 ​mg/kg + 1 ​mg/kg in combination treatments. One-way ANOVA and two tailed student t-test were used, followed by Tukey post-hoc analysis. Values are presented in the mean ​± ​SEM. ∗p ​< ​0.05, ∗∗p ​< ​0.01, ∗∗∗p ​< ​0.001, ∗∗∗∗p ​< ​0.0001.

While under our experimental conditions, the relative expression of CB_1_ transcripts in the hippocampus was relatively low and did not change between the control and the treated group, CB_2_R transcript expression was significantly increased in hippocampi from untreated SAMP8 if compared with samples from untreated SAMR1 mice (Fig. 3A). Remarkably, we found a significant reduction in CB_2_R gene expression in all SAMP8 mice that were treated with agonist and the G9a inhibitor (Fig. 3A). This reduction was more pronounced when the G9a inhibitor was administered, even in the absence of CBR agonists.

Considering that CB_2_Rs are upregulated in activated microglia and reactive astrocytes associated with the hallmarks of AD [[31], [32], [33], [34]] we examined several microglia and astroglia markers (Fig. 3B and C). First, compared with SAMR1 mice, we found in hippocampal samples of the SAMP8 animal significantly increased gene expression levels of *Trem2 and Cd33* and increased levels of *iNOS* while *Arg1* gene expression was reduced (Fig. 3B). Notably, some of the pharmacological treatments led to a reversal of the increase of *Trem2, Cd33,* and *iNOS* gene expression after single or dual treatment. In the case of *Arg1* gene expression, a significant reduction was only detected after JWH133 and UNC0642 cotreatment (Fig. 3B). These results show that hippocampal alterations detected in the model of accelerated senescence can be partially restored by G9a inhibition and/or CBR activation.

Regarding astroglia and pro-inflammatory markers, we analyzed *Gfap, Il-6,* and *Tnf-α,* which are typically elevated in AD patients [33,35,36]. As expected, we found increased gene expression of all markers in the SAMP8 compared to the SAMR1, with *Gfap* and *TNF-α* but not *Il-6* reaching statistical significance (Fig. 3C). A significant decrease of the altered levels of *Gfap* and *Tnf-α* gene expression was achieved by the pharmacological treatments, especially by UNC0642, the enzyme inhibitor (Fig. 3C).

### Modulation of immediate-early genes and enhanced neuronal repair following a single or dual therapeutic approach based on G9a inhibition or CBR activation in SAMP8

**Fig. 4:**
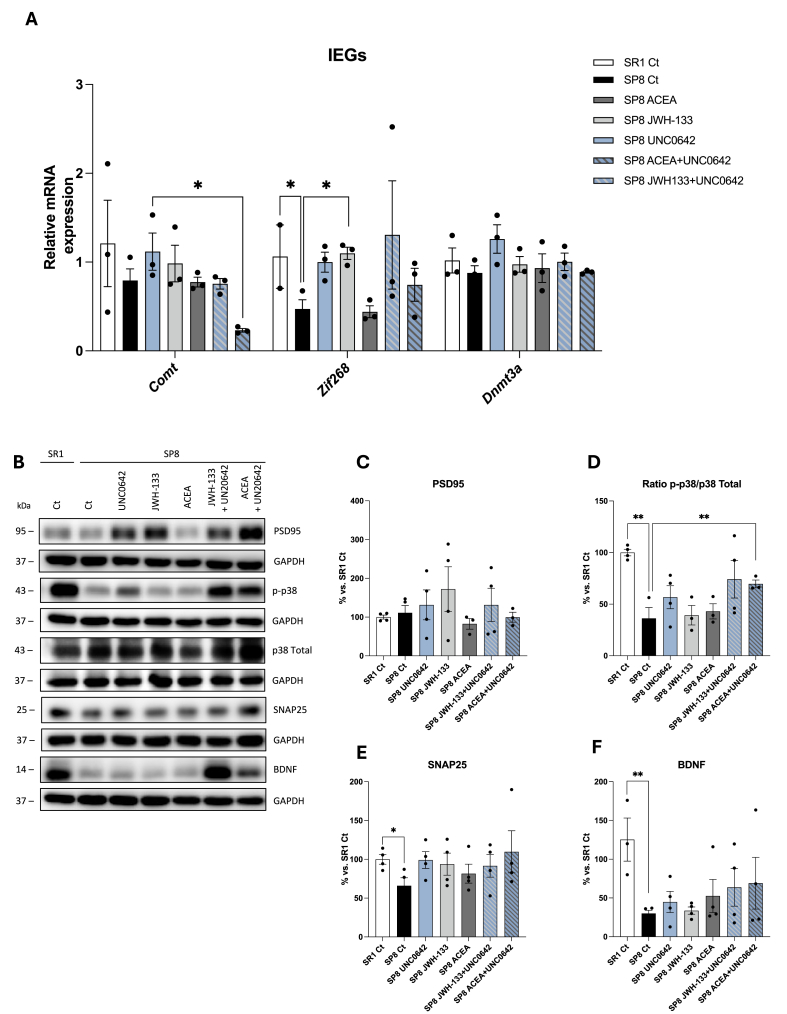
**Relative expression of transcripts coding for IEGs (A) and protein expression of IEGs (B**–**G).** mRNa isolation from hippocampi and qPCR assays are detailed in Methods. Samples were from 6-month-old SAMR1 untreated (n ​= ​3), (n ​= ​3), SAMP8 untreated (n ​= ​3) and SAMP8 treated with UNC0642 (n ​= ​3), JWH133 (n ​= ​3), ACEA (n ​= ​3), UNC0642 ​+ ​JWH133 (n ​= ​3) or UNC0642+ACEA (n ​= ​3), Dose was 1 ​mg/kg for the single treatment and 1 ​mg/kg ​+ ​1 ​mg/kg in combination treatments. One-way ANOVA and two-tailed student t-test were used, followed by Tukey post-hoc analysis. Values are presented in the mean ​± ​SEM. ∗p ​< ​0.05. **B.** Representative WBs for protein expression are shown in (B). Quantification of the expression of sAPPβ PSD95, p-p38 vs total p38 ratio, SNAP25, and BDNF is shown in panels **C to G.** Samples were from 6-month-old SAMR1 untreated (n ​= ​4), SAMP8 untreated (n ​= ​4) and SAMP8 treated with UNC0642 (n ​= ​4), JWH133 (n ​= ​3), ACEA (n ​= ​4), UNC0642 ​+ ​JWH133 (n ​= ​3) or UNC0642+ACEA (n ​= ​4). Dose was 1 ​mg/kg for the single treatment and 1 ​mg/kg + 1 ​mg/kg in combination treatments. Data in SAMR1 untreated are adjusted to 100 ​% for normalization. One-way ANOVA and two-tailed student t-test were used, followed by Tukey post-hoc analysis. Values are presented in the mean ​± ​SEM. ∗p ​< ​0.05, ∗∗p ​< ​0.01.

Expression of immediate-early genes (IEGs) changes rapidly in response to various pathophysiological changes, as in AD [37,38]. Here, we examined the expression of the genes for three IEGs, namely *Catechol-O-Methyltransferase* (*Comt*)*, Early Growth Response 1* (*Egr1*, also known as *Zif268*)*, and DNA Methyltransferase 3 Alpha* (*Dnmt3a*). While the level of transcripts for *Dnmt3a* was similar in samples from all groups, the pharmacological treatments reversed the altered expression of the other two genes in the SAMP8 mouse (Fig. 4A).

The soluble amyloid precursor protein beta (sAPPβ), a cleavage product of the amyloid precursor protein (APP), has been implicated in AD due to its role in the amyloidogenic pathway, which contributes to the formation of amyloid-beta plaques [19]. Although non-significant results were obtained, the pharmacological treatments reversed the altered sAPPβ protein levels found in samples from the SAMP8 mice (Fig. 4B and C). Loss of synapses in the hippocampus is another neuropathological hallmark of AD [39,40]. Therefore, we examined by immunoblotting the levels of a presynaptic SNAP25 and a postsynaptic PSD95 protein marker. The amount of PSD95 was not similar in both animal strains (Fig. 4D) and the level of SNAP25, which was reduced in the SAMP8 model, was restored by the pharmacological treatments (Fig. 4F). The level of the brain-derived neurotrophic factor (BDNF), which was markedly reduced in the SAMP8 model, was partially restored, especially in cotreatments with UNC0642 and any of the receptor agonists Fig. 4G. Finally, we examined the degree of phosphorylation of p38, a MAPK involved in regulating synaptic plasticity. The level of kinase phosphorylation was significantly lower in the SAMP8 animal, but treatment with UNC0642 and ACEA or JWH133 partially rescued the reduced p38 phosphorylation found in the hippocampus of SAMP8 mice (Fig. 4E). Overall, the results show the benefits of G9a inhibitor treatment, which were often more significant in dual inhibitor/agonist cotreatments.

### Improved synaptic plasticity and cognitive performance after G9a inhibition, cannabinoid receptor agonism, or dual therapy approach in SAMP8

**Fig. 5:**
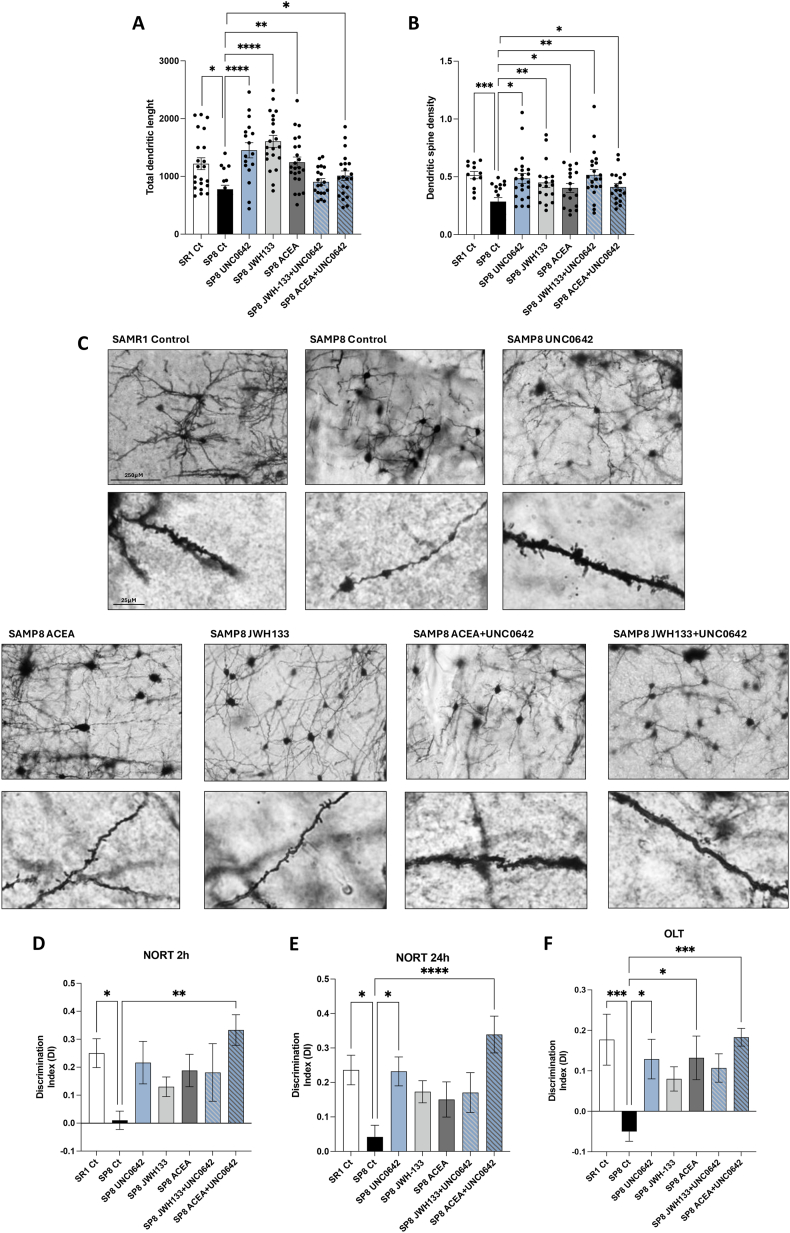
**Dendrite parameters and behavioral studies upon pharmacological treatment with CB receptor agonists and G9a inhibitor.** Quantification of the total dendritic length (A) and of dendritic spine density (B) in hippocampal samples. After the pharmacological Values are expressed as mean ​± ​SEM. Groups were compared using one-way ANOVA analysis followed by Tukey's post-hoc test. n ​= ​20–25 per group for neurons and spines. ∗p ​< ​0.05, ∗∗p ​< ​0.01, ∗∗∗p ​< ​0.001, ∗∗∗∗p ​< ​0.0001. Representative images of Golgi-stained neurons allowing the quantification of dendrite length and dendritic spine density are shown in **C**. (scale bar ​= ​250 ​μM and 25 ​μM, respectively). Panels **D to F** show discrimination index (DI) in behavioral assays consisting of NORT 2 ​h, NORT 24 ​h and OLT. Experiments were conducted on 6-month-old SAMR1 untreated (n ​= ​10), SAMP8 untreated (n ​= ​10) and SAMP8 treated with UNC0642 (n ​= ​7), JWH133 (n ​= ​10), ACEA (n ​= ​8), UNC0642 ​+ ​JWH133 (n ​= ​8) or UNC0642+ACEA (n ​= ​9). Dose was 1 ​mg/kg for the single treatment and 1 ​mg/kg + 1 ​mg/kg in combination treatments. One-way ANOVA and two-tailed student t-test were used for statistical analysis. Values are presented in the mean ​± ​SEM. ∗p ​< ​0,05, ∗∗p ​< ​0,01, ∗∗∗p ​< ​0,001.

Synaptic dysfunction and synapse loss are key features of AD [41]. Golgi staining was undertaken to investigate whether G9a inhibition, CBR activation or dual treatments could modulate the density of dendritic spines and dendrite length. As expected, there was a significant reduction in branching length and dendritic spine density in the hippocampus of the SAMP8 mice compared to the hippocampus of the SAMR1 animal (Fig. 5A–C). In agreement with the above-described benefits observed in biochemical and gene expression parameters, the G9a inhibitor with or CBR agonists restored, totally or partially, the deficits found in the SAMP8 animal at the dendritic level. In the case of dendritic length, all treatments lead to a complete recovery, with single treatments leading to an increase over those found in the hippocampus of the SAMR1 animal (Fig. 5A). Regarding spine density, the most efficacious treatments leading to levels similar to those in SAMR1 mice were obtained with UNC0642 or dual UNC0642/JWH133 treatment (Fig. 5B and C).

Cognitive performance was assessed using the NORT and OLT tasks to evaluate working and spatial memory in SAMP8 mice. Untreated SAMP8 animals significantly reduced the discrimination index (DI) for both short- and long-term memory compared to untreated SAMR1 controls (Fig. 5D and E). Treatment interventions revealed significant recovery in short-term memory, with the dual UNC0642/ACEA treatment demonstrating the highest efficacy (Fig. 5D and E). In the OLT task, all treatments resulted in partial recovery of spatial memory, except for the dual UNC0642/ACEA treatment, which achieved complete recovery (Fig. 5F). These findings indicate that the combined inhibition of G9a and activation of CB_1_R effectively mitigates cognitive decline in SAMP8 mice.

## Discussion

This study provides compelling evidence for the neuroprotective effects of G9a inhibition combined with cannabinoid receptor activation in AD, offering a multifaceted therapeutic approach to address cognitive decline and neurodegeneration. By integrating in vitro and in vivo approaches, we shed light on the complex interplay between epigenetic regulation and the ECS, emphasizing this dual intervention's mechanistic and therapeutic implications. We also demonstrated a potential synergistic effect for critical endpoints in the study of new therapeutic approaches in AD treatment, suggesting, in part, the dual approach presented in this study. Specifically, this synergistic interaction between G9a inhibition (via UNC0642) and CB_1_R activation (via ACEA) arises from complementary molecular mechanisms that enhance neuroprotective signaling pathways while mitigating neuroinflammation and synaptic dysfunction in AD models.

Moreover, the selection of dosing regimens for UNC0642, ACEA, and JWH133 in this study was informed by prior preclinical investigations, with adjustments to suboptimal levels to unmask potential synergistic interactions. For UNC0642, acute doses of 5 ​mg/kg (i.p.) have demonstrated robust target engagement in vivo, achieving a plasma Cmax of 947 ​ng/mL and suppressing H3K9me2 levels in xenograft models [22]. Chronic administration at 2–4 ​mg/kg/day was well-tolerated in Prader-Willi syndrome (PWS) mouse models, with sustained epigenetic modulation and no significant toxicity [42]. We selected a reduced dose of 1 ​mg/kg to avoid maximal inhibition of G9a, thereby allowing detection of combinatorial effects with CB_1_R activation.

Regarding the CB_1_R agonist ACEA, doses of 0.5–1.5 ​mg/kg (low) and 5 ​mg/kg (mid) have been validated in neuroprotection studies. In ischemic stroke models, 1 ​mg/kg ACEA improved motor recovery and reduced neuronal death without inducing hyperactivation [43]. Similarly, studies in aggressive behavior models demonstrated that 1–2 ​mg/kg ACEA modulated CB_1_R-mediated neurotransmission without adverse effects [44]. Our use of 1 ​mg/kg aligned with these subthreshold doses to isolate synergistic interactions rather than standalone efficacy.

The CB_2_R agonist JWH133 was administered at 1 ​mg/kg, a dose below the 5 ​mg/kg mid-range used in prior addiction and neuroinflammation studies [45,46]. For instance, 1–2 ​mg/kg JWH133 reduced cocaine self-administration in mice without altering baseline locomotion, while 0.25–0.5 ​mg/kg improved cognitive performance in memory tasks. Higher doses (e.g., 20 ​mg/kg) induced pronounced anti-inflammatory effects in neurodegenerative models [46], but such levels risk masking synergistic contributions. By selecting 1 ​mg/kg, we prioritized detection of combinatorial signaling over individual receptor saturation.

This dosing strategy-employing submaximal concentrations of UNC0642 (1 ​mg/kg), ACEA (1 ​mg/kg), and JWH133 (1 ​mg/kg)-enabled the identification of synergistic neuroprotective and anti-inflammatory effects that would otherwise be obscured at higher, fully efficacious doses. The approach is consistent with methodologies in polypharmacology, where subthreshold dosing reveals emergent interactions between pathways [47]. Furthermore, the tolerability of these doses is supported by prior pharmacokinetic and toxicity profiles, ensuring that observed effects reflect mechanistic synergy rather than off-target toxicity.

First, we examined the modulation of CBR signaling by G9a in HEK-293T cells and primary hippocampal neuron cultures. Interestingly, our findings offer insights into the complex interplay between epigenetic regulation by G9a and the endocannabinoid system. Our results demonstrate that G9a inhibition selectively modulates CB_1_R signaling while leaving CB_2_R activity largely unaffected. This specificity is particularly relevant, as it provides a means to enhance CB1R-mediated neuroprotection while potentially mitigating the psychoactive side effects associated with direct CB_1_R agonists. Additionally, we investigated the impact of G9a inhibition with UNC0642 and selective CB_1_R (ACEA) and/or CB_2_R (JWH133) agonists on cognitive decline and neuroinflammation in the SAMP8, a late-onset Alzheimer's disease (LOAD) mouse model [[48], [49], [50]]. In hippocampal neurons, the potentiation of CB_1_R signaling by G9a inhibition was evident through enhanced ERK1/2 phosphorylation and cAMP signaling, indicating a downstream amplification of neuroprotective pathways. Thus, the differential effects observed with single and dual treatments provide critical mechanistic insights into the interplay between epigenetic regulation and cannabinoid signaling in AD. These results expand on previous observations, such as those by Bellver-Sanchis et al. (2024) [15], by highlighting the regulatory role of G9a in ECS signaling, specifically in neuronal contexts. Therefore, this strategy offers a promising avenue for addressing cognitive deficits in AD while potentially avoiding some psychoactive effects associated with direct CB_1_R agonism [51,52].

Regarding the cognitive results, we showed a cognitive recovery observed in SAMP8 mice treated with UNC0642 and ACEA, particularly in spatial memory (OLT task), underscoring this dual approach's therapeutic efficacy. These results are significant, as they demonstrate not only behavioral recovery but also molecular changes derived from these pharmacological interventions. The chronic inflammatory process in neurodegeneration and AD has been identified as a primary contributor to disease progression [53]. In addition, microglia activation is a crucial event in neuroinflammation and neurodegeneration [[54], [55], [56]]. Notably, the ECS plays a role in the microglia phenotype. For this reason, we evaluated CB_1_R and CB_2_R markers in SAMP8 and SAMR1, the healthy control animal as well as after single or dual treatments. Thus, the pronounced reduction in CB_2_R gene expression across treated groups, particularly with G9a inhibition and dual treatments, further supports the hypothesis that targeting G9a modulates microglial activation states, as CB_2_R is typically upregulated in reactive glia associated with AD pathology [57]. Notably, the distinct patterns of modulation observed with single treatments (ACEA and JWH133) versus dual treatments highlight the complexity of neuroinflammatory processes in AD. These findings align with prior studies indicating that neuroinflammation is a key driver of AD progression, highlighting the dual anti-inflammatory and neuroprotective roles of this therapeutic strategy. The results are particularly significant as they demonstrate both behavioral recovery and a reduction in hallmark neuroinflammatory markers such as TREM2, CD33, and iNOS.

On the other hand, synaptic plasticity plays a crucial role in age-related cognitive decline and AD [58,59]. Furthermore, AD is associated with synaptic function and impairment of plasticity, particularly in the hippocampus [[52], [53], [54]]. Our results prove that G9a inhibition and ECS activation can synergistically promote synaptic repair. Importantly, we found restoration of dendritic spine density and branching length, as revealed by Golgi staining, which reflects a reversal of structural deficits, delivering an exciting improvement in neuronal function in the SAMP8 hippocampus. Additionally, the recovery of synaptic markers such as SNAP25 and PSD95, along with increased BDNF expression, suggests that these interventions target both pre- and postsynaptic mechanisms critical for plasticity. The partial recovery of p38 phosphorylation further implicates MAPK signaling in mediating these neuroprotective effects. These findings resonate with previous studies, such as those by Griñán-Ferré et al. [23], which identified synaptic plasticity enhancement as a critical outcome of G9a inhibition.

On the other hand, the differential effects observed between single and dual treatments emphasize the complexity of the mechanisms underlying their neuroprotective actions. While CB_1_R activation may promote neuronal survival and plasticity via the BDNF/TrkB/Akt pathway, CB_2_R modulation likely exerts immunomodulatory effects through TLR4/NF-κB signaling. These assumptions are supported by several studies [51,57,60,61]. G9a inhibition appears to act upstream of these pathways, orchestrating a synergistic response that addresses neuroinflammatory and synaptic deficits [15]. The ability of UNC0642 to potentiate these effects when combined with ECS agonists highlights the therapeutic potential of a multi-target approach, particularly in diseases with multifactorial pathologies like AD (Fig. 5).

Moreover, future studies should explore the long-term effects of these treatments and investigate potential side effects or tolerance development. Additionally, examining the impact of these treatments on different AD hallmarks would provide a more comprehensive understanding of their potential to modify disease progression. Recent work by Rothammer et al. (2022) [62] has shown that G9a inhibition can reduce oxidative stress and promote neuronal survival, suggesting a potential role in modifying AD progression. These additional studies in AD mouse models further support the potential of combining G9a inhibition with cannabinoid receptor modulation, specifically CB_1_R, as a promising therapeutic approach for AD. Findings across different models and treatment paradigms underscore the robustness of this multi-target strategy in addressing various aspects of AD pathology.

## Conclusion

This study underscores the therapeutic promise of combining G9a inhibition with targeted cannabinoid receptor modulation as a multifaceted approach to AD. G9a inhibition epigenetically primes the neural environment by reversing repressive histone methylation, thereby restoring CB_1_R expression and enhancing the efficacy of CB_1_R-mediated neuroprotection. At the same time, G9a inhibition upregulates neuroprotective factors, further reducing neuroinflammation and supporting synaptic resilience. When paired with selective activation of CB_1_R-which directly counters AD-related synaptic and inflammatory deficits-and careful modulation of CB_2_R to curb excessive inflammation, this dual strategy addresses the major pathological hallmarks of AD: cognitive decline, neuroinflammation, and synaptic dysfunction. By simultaneously targeting these interconnected pathways, this approach offers a more comprehensive and robust therapeutic framework than single-pharmacological interventions, paving the way for innovative treatments that more effectively combat the complexity of aging-related neurodegenerative diseases.

## Authors contribution

Conceptualization, C.G.-F.; methodology, A.B.-S., G.N., M.R.-V., D.O.-S., R.F., M.P., J.L. and C.G.-F.; Analyzed the data and drafted the manuscript, J.L., G.N., A.B.-S., M.R.-V., R.F., M.P. and C.G.-F.; writing—original draft preparation, A.B.-S., G.N., R.F., D.O.-S., M.P., and C.G.-F.; writing—review and editing, A.B.-S., G.N., R.F., D.O.-S., M.P., and C.G.-F.; supervision, C.G.-F. All authors have read and agreed to the published version of the manuscript.

## Declaration of competing interest

The authors declare that they have no known competing financial interests or personal relationships that could have appeared to influence the work reported in this paper.
