# Supplementary material for: Neuroprotective effects of G9a inhibition and cannabinoid receptor activation in Alzheimer's disease through a pharmacological approach
Source: Neurotherapeutics. 2025 May 31;22(5):e00616. doi: 10.1016/j.neurot.2025.e00616 (PMC12491791; doi:10.1016/j.neurot.2025.e00616)
Supplement: Multimedia component 1 [file mmc1.docx]

**Table 1. Antibodies**

| Antibody | Host | Catalog | WB dilution |
| --- | --- | --- | --- |
| sAPPβ | Rabbit | Covance/SIG-39139 | 1:500 |
| PSD95 | Rabbit | Gene Tex/133091 | 1:1000 |
| p-p38 | Mouse | Santa Cruz/ sc-166182 | 1:500 |
| p38 | Mouse | Santa Cruz/ sc-81621 | 1:1000 |
| SNAP25 | Mouse | Santa Cruz/ sc-20038 | 1:1000 |
| BDNF | Mouse | Bios/ BS-4989R | 1:1000 |
| GAPDH | Mouse | Millipore/MAB374 | 1:2000 |
| Goat-anti-mouse Alexa Fluor Plus 680 |  | Life Technologies/A32729 | 1:2000 |
| Goat-anti-rabbit Alexa Fluor Plus 800 |  | Life Technologies/ A32735 | 1:2000 |

| Gene | Forward sequence (5-3’) | Reverse sequence (5-3’) |
| --- | --- | --- |
| *Arg1* | CTCCAAGCCAAAGTCCTTAGAG |  |
| *Trem2* | TTGCTAACCTGACACCCTTTG | CGGTGCAGGTTGAGCATGTA |
| *Cd33* | CGGCAGCATCAAATGTTTCAG | AACTGGCAGGTAGAAGGCAACTC |
| *iNOS* | CTGCACTTAACCTGGCATATCCA | GGCTCCAGCAGGTGAGAGAA |
| *Gfap* | GCCCCCAGCAGTGATTCATA | GGAACCCATGTAGGCAGCTT |
| *Il-6* | CCCGTAGACCTAGGGAGGAC | CAATACACTCCATGCGGTTG |
| *Tnf-a* | CCGAACCCCATATCGTCTGAA | TGTTCCCCTTTGCCTCCCTTC |
| *CB1* | TGCGAGTATTACCTCCGCCAT | TCACGTGCTCAAAAGTGTCAG |
| *CB2* | GGAGCGCATCGAGTGACTT | CCTCACTGCGGCCAGTATAG |
| *Comt* | GTCAGACCCATAGCCTCCC | CTCGGACTGAAATCTCGAAGTTC |
| *Zif268* | CACCTACCTGAGAAGGAATTTGC | GACCAGGTGCCTTGGGATAC |
| *Dnmt3a* |  |  |
| *β-actin* | CAACGAGCGGTTCCGAT | GCCACAGGTTCCATACCCA |
